# BSE Case Associated with Prion Protein Gene Mutation

**DOI:** 10.1371/journal.ppat.1000156

**Published:** 2008-09-12

**Authors:** Jürgen A. Richt, S. Mark Hall

**Affiliations:** 1 National Animal Disease Center, United States Department of Agriculture, Agriculture Research Service, Ames, Iowa, United States of America; 2 National Veterinary Services Laboratories, Pathobiology Laboratory, Animal and Plant Health Inspection Service, United States Department of Agriculture, Ames, Iowa, United States of America; University of Alberta, Canada

## Abstract

Bovine spongiform encephalopathy (BSE) is a transmissible spongiform encephalopathy (TSE) of cattle and was first detected in 1986 in the United Kingdom. It is the most likely cause of variant Creutzfeldt-Jakob disease (CJD) in humans. The origin of BSE remains an enigma. Here we report an H-type BSE case associated with the novel mutation E211K within the prion protein gene (*Prnp*). Sequence analysis revealed that the animal with H-type BSE was heterozygous at *Prnp* nucleotides 631 through 633. An identical pathogenic mutation at the homologous codon position (E200K) in the human *Prnp* has been described as the most common cause of genetic CJD. This finding represents the first report of a confirmed case of BSE with a potential pathogenic mutation within the bovine *Prnp* gene. A recent epidemiological study revealed that the K211 allele was not detected in 6062 cattle from commercial beef processing plants and 42 cattle breeds, indicating an extremely low prevalence of the E211K variant (less than 1 in 2000) in cattle.

## Author Summary

Bovine spongiform encephalopathy (BSE or Mad Cow Disease), a transmissible spongiform encephalopathy (TSE) or prion disease of cattle, was first discovered in the United Kingdom in 1986. BSE is most likely the cause of a human prion disease known as variant Creutzfeldt Jakob Disease (vCJD). In this study, we identified a novel mutation in the bovine prion protein gene (*Prnp*), called E211K, of a confirmed BSE positive cow from Alabama, United States of America. This mutation is identical to the E200K pathogenic mutation found in humans with a genetic form of CJD. This finding represents the first report of a confirmed case of BSE with a potential pathogenic mutation within the bovine *Prnp* gene. We hypothesize that the bovine *Prnp* E211K mutation most likely has caused BSE in “the approximately 10-year-old cow” carrying the E221K mutation.

## Introduction

Transmissible spongiform encephalopathy (TSE) agents induce fatal neurodegenerative diseases in humans and in some mammalian species [1]. According to the prion-only hypothesis, infectious prions are composed of an abnormal isoform of a host-encoded glycoprotein, called prion protein (PrP^c^). The disease-associated form, PrP^d^, is derived from PrP^c^ by a post-translational mechanism that involves conformational change [1]. Human TSEs include Creutzfeldt–Jakob disease (CJD), Gerstmann–Sträussler–Scheinker syndrome, Kuru and Fatal Familial Insomnia [1]. In animals, several distinct TSE diseases are recognized: Scrapie in sheep and goats, transmissible mink encephalopathy in mink, chronic wasting disease in cervids, and bovine spongiform encephalopathy (BSE) in cattle [2]. BSE was first detected in 1986 in the United Kingdom and is the most likely cause of variant CJD (vCJD) in humans. The origin of the original case(s) of BSE still remains an enigma. Hypotheses include (i) sheep- or goat-derived scrapie-infected tissues included in meat and bone meal fed to cattle, (ii) a previously undetected sporadic or genetic bovine TSE contaminating cattle feed or (iii) origination from a human TSE through animal feed contaminated with human remains [3]. This study will provide support to the hypothesis that BSE originated from a previously undetected genetic bovine TSE contaminating cattle feed in the U.K.

## Results

Here we report a case of bovine BSE associated with a mutation within the prion protein gene (*Prnp*) sequence, not previously described for the bovine *Prnp*. The animal (called “U.S. BSE Alabama”) was an approximately 10 year-old red crossbred (*Bos indicus*×*Bos taurus*) hybrid beef cow from Alabama (see Materials and Methods). The ELISA-based BSE test (see Materials and Methods) on brainstem from this animal was repeated five times and revealed a strongly positive reaction with mean optical density (OD) value of 2.40±0.57, whereas the OD value of bovine control obex was <0.04.

**Figure 1:**
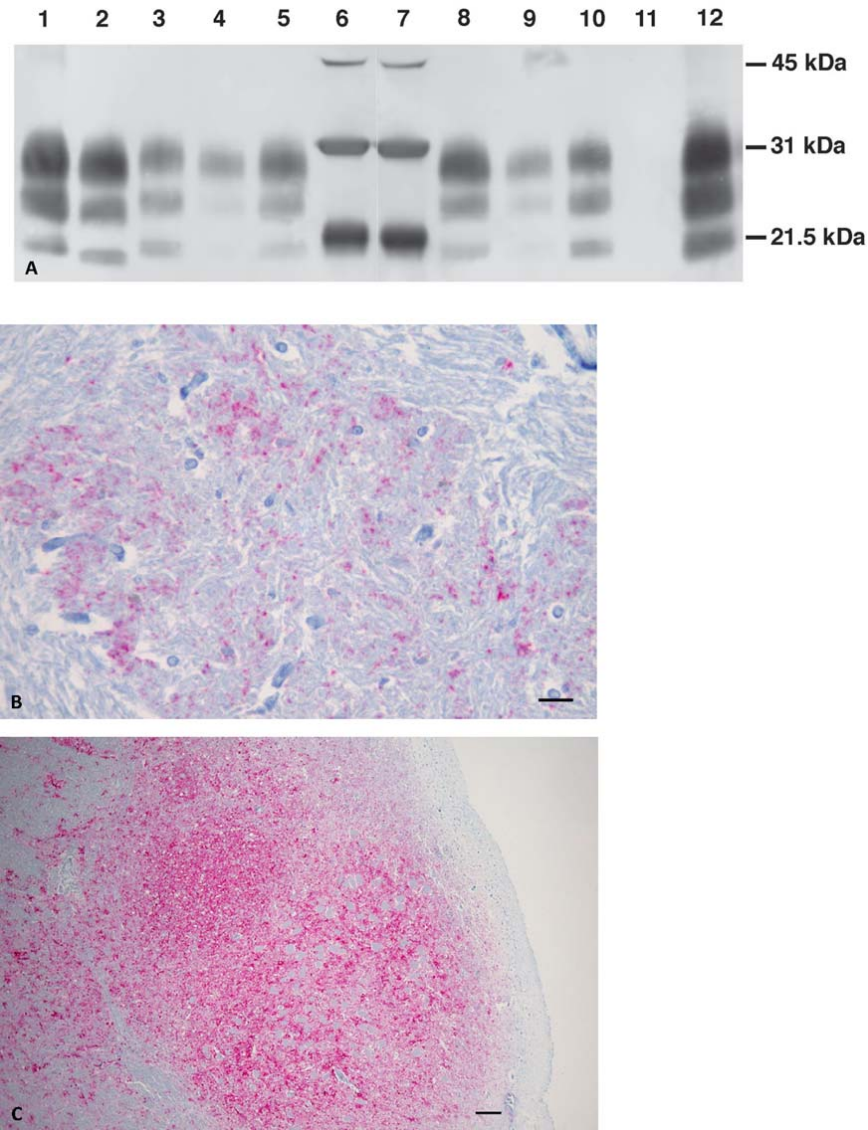
Analysis of brainstem samples from BSE-infected animals employing various methods. (A) Hybrid Immunoblot Analysis using enriched samples: Lanes 1–5: monoclonal antibody 6H4 (raised against human PrP residues 144–152), lanes 8–12 monoclonal antibody P4 (raised against ovine PrP residues 89–104): 1 = sheep scrapie control, 2 mg; 2 = classical BSE (2003 U.S. BSE case), 2 mg; 3 = H-type BSE case (2004 U.S. BSE case), 2 mg; 4 = U.S. BSE Alabama case, 1 mg; 5 = U.S. BSE Alabama case, 2.5 mg; 6,7 = protein weight maker; 8 = U.S. BSE Alabama case, 2.5 mg; 9 = U.S. BSE Alabama case, 1 mg; 10 = H-type BSE case (2004 U.S. BSE case), 2 mg; 11 = classical BSE (2003 U.S. BSE case), 2 mg; 12 = sheep scrapie control, 2 mg. (B) Immunohistochemistry of the U.S. BSE Alabama case (H-type BSE) using PrP-specific monoclonal antibody F99/97.6.1. Brainstem at the level of obex was examined. Bar = 35 µm. (C) Immunohistochemistry of a classical BSE case [4] using PrP-specific monoclonal antibody F99/97.6.1. Brainstem at the level of obex was examined. Spongiform changes are found in the area with highly PrP^d^-positive cells. Bar = 90 µm.

Confirmatory BSE tests employing Western Blot (WB) and immunohistochemical (IHC) analyses for presence of PrP^d^ were subsequently performed (see Materials and Methods). Immunoblots (Figure 1A) revealed (i) presence of PrP^d^ and (ii) intensity of reaction with antibody P4 similar as with antibody 6H4 at identical milligram equivalent amounts. This reaction pattern was described as being unusual or atypical for BSE [4]. Molecular weight analysis revealed unglycosylated and monoglycosylated isoforms of PrP^d^ migrated with an apparent molecular weight higher than respective isoforms of classical or C-type BSE isolates (Figure 1A). A similar migration pattern was observed for the U.S. BSE 2004 isolate, an H-type BSE isolate [4]. Obvious lesions of spongiform encephalopathy diagnostic for BSE were not present in the brainstem, however it was positive for the presence of PrP^d^ by IHC (Figure 1B). Distribution of PrP^d^ in the brainstem of this animal was not as uniform or as intense as seen with the C-type U.S. BSE case from 2003 (Figure 1C) [4]. We concluded from these studies, that this animal contracted an H-type BSE phenotype. Recent work on the molecular characterization of cattle PrP^d^ has allowed to define criteria for the identification of atypical BSE cases in cattle, which showed molecular features of the PrP^d^ distinct from the majority of cattle with C-type BSE [4]–[6]. There have been two molecular types of unusual BSE isolates described in the literature [5],[6]: (i) a type with a lower molecular mass of the unglycosylated isoform (L-type) and (ii) a type with higher molecular mass of the unglycosylated isoform (H-type) when compared to C-type of BSE.

**Figure 2:**
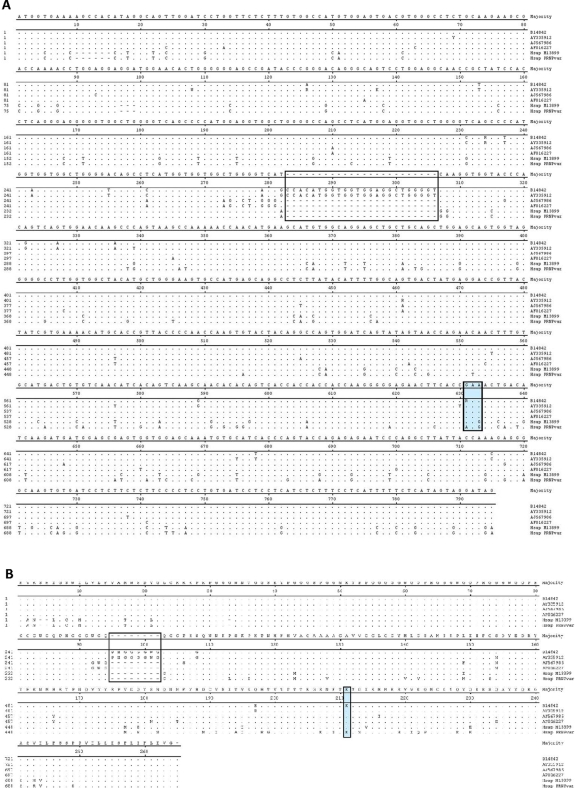
Alignment of bovine, ovine, cervid and human *Prnp* sequences. (A) Nucleotide sequences. Standard single letter codes are used for nucleotides. Y = C or T; R = A or G; K = G or T; W = A or T. Boxed area indicates the 6^th^ octapeptide-repeat of the bovine protein (U.S. BSE Alabama case and sequence AY335912). Additional *Prnp* sequences are as follows: AJ567986 (sheep), AF016227 (elk), Hsap M13899 (human, normal) and Hsap PRNPvar [human, variant; see [13]]. (B) Amino acid sequences. Standard IUPAC single letter codes are used for amino acids. Codon numbering refers to the most common six-copy octapeptide repeat allele for *Bos Taurus*. Boxed area indicates the 6^th^ octapeptide repeat of the bovine protein [animals B14842 [4] and AY335912]. AJ567986 (sheep), AF016227 (elk), Hsap M13899 (human, normal) and Hsap PRNPvar [human, variant; see [13]] each contain a 5 octapeptide repeat region in the protein.

In order to confirm the specimen from this case was of cattle origin and to determine whether the case was associated with a *Prnp* mutation, the full coding sequence from exon 3 of the *Prnp* was amplified from DNA isolated from fresh brainstem material (see Materials and Methods) and aligned with known *Prnp* sequences from cattle, sheep and cervids (Figure 2A,B). The *Prnp* DNA sequence of this animal is of bovine origin, different from sheep and cervid *Prnp* sequences (Figure 2A). The sequence was heterozygous on two positions: a synonymous polymorphism on codon 78 (CAA/CAG; Q78Q) and a non-synonymous polymorphism on codon 211 (GAA/AAA; E211K; Figure 2A,B; Table 1). The animal had six copies of the octapeptide repeat region on both of its *Prnp* alleles (Figure 2A,B). The finding of the E211K mutation is of significant interest because an identical mutation, E200K, at the homologous codon 200 position in human *Prnp* (Figure 1D) has been described as the most common mutation in humans with genetic CJD [7].

**Table 1:** DNA sequence analysis of codon 211 of the bovine *Prnp* and codon 200 of the human *Prnp*.

	Nucleotide	Amino Acid
**Bovine majority**	G A A/G A A	E211/E211
**U.S. 2006 BSE case**	G A A/A A A	E211/K211
**Human majority**	G A G/G A G	E200/E200
**Human genetic CJD**	G A G/A A G	E200/K200

## Discussion

Our results demonstrate for the first time a potential pathogenic mutation (E211K) within the *Prnp* gene of a bovine with an H-type BSE phenotype at a position representing the most common mutation in humans (E200K) associated with genetic TSEs [7]. This mutation was not found in the *Prnp* gene of other North American (1 H-type U.S.; 1 H-type and 1 L-type Canadian) and European (7 H-type and 3 L-type cases) cattle [8] and a miniature zebu (H-type) [9] with atypical BSE phenotypes. The functional significance of this finding, however, remains unknown. Importantly, the penetrance of the E200K mutation in humans is very high [7],[10]. The origin of atypical BSE cases still remains unexplained. Several hypotheses have been considered including the existence of a previously unrecognized “sporadic” form of a TSE in this species. The detection of the E211K *Prnp* mutation, known to be pathogenic in humans, in a 10 year old hybrid cow (*Bos indicus*×*Bos taurus*) with H-type BSE could provide additional support to the following hypotheses: (i) that U.K. BSE has been acquired from a genetic case or cases of cattle BSE, (ii) that all three etiological forms of human TSEs (sporadic, genetic and infectious) are also present in cattle, and (iii) that BSE started on the Indian subcontinent. However, more data are required to support these hypotheses. It is well known, that large amounts of mammalian protein material were imported from India to the U.K. during the relevant time period (late 1970s and early 1980s) [3]. Therefore it could be speculated that one possible route of contamination of U.K. cattle with BSE was through animal feed containing imported meat and bone meal material contaminated with a case or cases of genetic BSE.

Epidemiological investigations conducted by USDA personnel failed to reveal any evidence of a feed source contaminated with TSE material fed to this animal (http://www.aphis.usda.gov/newsroom/hot_issues/bse/downloads/EPI_Final5-2-06.pdf). There are several possibilities for the origin of the *Prnp* K211 allele in animal B14842: (i) it arose *de novo* in a germ line cell from the U.S. BSE Alabama animal or one of its parents; (ii) it arose as a somatic mutation in the U.S. BSE Alabama animal (rather unlikely), and (iii) it is present in a cattle population or breed yet to be found. Recently, it was determined that the 2-year-old heifer offspring of the U.S. BSE Alabama cow also carries the E211K polymorphism, indicating that the allele is heritable and may persist within the cattle population (11) In a recent epidemiological study which included 6062 cattle from 5 commercial beef processing plants (3892 carcasses) and 2170 registered cattle from 42 breeds., the K211 allele was not detected using a newly developed mass spectrometry assay specific for the E211K variant [12]. These data indicate a rather low prevalence of the E211K variant of less than 1 in 2000 cattle when using Bayesian analysis [12]. This newly developed assay system for K211 [12] will offer the possibility for genetic surveillance of cattle for rare pathogenic mutations that may be associated with BSE.

## Materials and Methods

### Animals

The animal B14842 (called U.S. BSE Alabama case), approximately 10 years old as determined by dentition, was a red crossbred (*Bos indicus*×*Bos taurus*) hybrid beef cow found in lateral recumbency on a farm in Alabama. She was euthanized by the attending veterinarian, the brainstem removed for BSE testing and shipped to the Georgia Veterinary Medical Diagnostic Laboratory, where it was found to be positive by a rapid enzyme-linked immunosorbent assay (ELISA)-based BSE test. The brainstem was then forwarded to the National Veterinary Services Laboratories in Ames, IA, USA, for confirmatory testing. The classical BSE case was reported previously [4].

### ELISA test

The rapid BSE test used in the U.S. is the Platelia/TeSeE™ ELISA BSE test (Bio-Rad, Hercules, CA, USA). Fresh samples from the brainstem were used for the analysis and the samples were treated with proteinase K (PK) in order to digest the PK-sensitive normal prion protein, PrP^c^.

### Imunoblot Analysis

Brain homogenates from the U.S. BSE Alabama case (animal B14842) were prepared from 1.1 gram of brainstem material and analyzed using the Prionics®-Check Western Kit (Prionics, Schlieren, Switzerland) with modifications and the OIE-recommended Scrapie Associated Fibril (SAF)-Immunoblot method (http://www.oie.int/eng/normes/mmanual/A_summry.htm). Preparation and analysis of brainstem homogenate using both methods has been described previously [4]. Please note that the brain homogenates were treated with PK (2 U/ml) for 60 minutes at 37°C before Western Blot analysis in order to digest the PK-sensitive PrP^c^. As positive control samples, BSE-positive brain material from the U.S. BSE cases 2003 and 2004 as well as a sheep scrapie isolate were used. As negative controls, brain material from a BSE-negative cow was used.

### Immunohistochemistry

Brain tissue was placed in 10% buffered formalin and after a minimum of 4 days of fixation appropriate sections of brainstem in the obex region were put in cassettes and kept in fresh formalin until they were processed for routine paraffin embedding. The procedure was described in detail previously [4]. The IHC results were interpreted as follows: (i) positive for PrP^d^: pink to red and (ii) background and negative for PrP^d^: only blue background. As positive controls, slides from the brainstem of a BSE-positive cow, obtained from the United Kingdom and from the U.S. BSE Case 2003 were used. As negative controls, slides from brainstem material of BSE-negative cattle and scrapie-negative sheep were used.

### DNA isolation, PCR amplification and sequence analysis

Genomic DNA was extracted from 200 µl of a 10% brain homogenate as described previously [4]. The fragment was sequenced in duplicate using the original two primers and two internal primers (available upon request) for a total of 8 reactions. Databases were searched using standard nucleotide-nucleotide BLAST at the National Center for Biotechnology Information Web Site (http://www.ncbi.nlm.nih.gov). The database is a collection of sequences from several sources, including GenBank and Reference Sequence. The nucleotide sequences of the *Prnp* gene of the U.S. BSE Alabama case was aligned using both CLUSTAL V and CLUSTAL W with the following GENBANK accession numbers: AY335912 (bovine), AY367641 (bovine), AF016227(elk), AY275712 (white-tailed deer), AF166334 (sheep), AJ567986 (sheep), and the Canadian BSE case [12] using Lasergene version 5.07 software (DNASTAR-Madison WI).

### Accession number

The GenBank accession number for the *Prnp* gene of U.S. BSE Alabama case is EU809428.
